# The appearance of renal cells cytoplasmic degeneration and nuclear destruction might be an indication of GNPs toxicity

**DOI:** 10.1186/1476-511X-10-147

**Published:** 2011-08-22

**Authors:** Mohamed Anwar K Abdelhalim, Bashir M Jarrar

**Affiliations:** 1 Department of Physics and Astronomy, College of Science, King Saud, University, P.O. Box 2455, Riyadh-11451, Saudi Arabia; 2 College of Applied Medical Sciences, Al-Jouf University, Saudi Arabia

**Keywords:** gold nanoparticles, renal tissue, histology, hydropic degeneration, nanotoxicity

## Abstract

**Background:**

Advances in nanotechnology have identified promising candidates for many biological and biomedical applications. Since the properties of nanoparticles (NPs) differ from that of their bulk materials, they are being increasingly exploited for medical uses and other industrial applications. The histological and the histochemical alterations in the renal tissues due to gold nanoparticles (GNPs) have not well documented and have not yet been identified. The aim of the present study was to investigate the particle-size effect of GNPs on the renal tissue in an attempt to address their potential toxicity.

**Methods:**

A total of 70 healthy male Wistar-Kyoto rats were exposed to GNPs received 50 or 100 μl of GNPs infusion of size (10, 20 and 50 nm for 3 or 7 days) to investigate particle-size effect of GNPs on the renal tissue. Animals were randomly divided into groups, 6 GNPs-treated rats groups and one control group. Groups 1, 2 and 3 received infusion of 50 μl GNPs of size 10 nm (3 or 7 days), size 20 nm (3 or 7 days) and 50 nm (3 or 7 days), respectively; while groups 4, 5 and 6 received infusion of 100 μl GNPs of size 10 nm, size 20 nm and 50 nm, respectively.

**Results:**

The histological alterations were mainly seen in the cortex and the proximal renal convoluted tubules were more affected than the distal ones. In comparison with respective control rats, exposure to GNPs doses has produced the following renal tubular alterations: cloudy swelling and renal tubular necrosis. Interstitial alterations included: intertubular blood capillaries dilatation, intertubular hemorrhage and inflammatory cell infiltrations. The glomeruli showed moderate congestion with no hypercelluraity and mesangial proliferation or basement membrane thickening.

**Conclusions:**

The induced histological alterations might be an indication of injured renal tubules due to GNPs toxicity that become unable to deal with the accumulated residues resulting from metabolic and structural disturbances caused by these NPs. These alterations were size-dependent with smaller ones induced more effects and related with time exposure of GNPs. The produced histological alterations may suggest that GNPs interact with proteins and enzymes of the renal tissue interfering with the antioxidant defense mechanism and leading to reactive oxygen species (ROS) generation which in turn may induce stress in the renal cells to undergo atrophy and necrosis. More histomorphologcal investigations are needed to address the potential threat of GNPs as a therapeutic and diagnostic tool.

## Introduction

Nanoparticles are an intermediate state of matter somewhere between bulk and molecular level. These particles have important application for cell labeling and imaging, drug delivery, biological sensors, diagnostic and therapeutic purposes mainly in cancer and photodynamic therapy [1-6]. Studies revealed that the NPs were rapidly taken into the system with the highest accumulation in the liver, spleen, lungs, aorta, esophagus and olfactory bulb [7]. Moreover, particles of nano-dimension are believed to be more biologically reactive than their bulk counter parts due to their small size and larger surface area to volume ratio [7,8].

Gold in its bulk form has long been considered an inert, noble metal with some therapeutic and even medicinal value hence GNPs are thought also to be relatively non-cytotoxic [9]. Yet there are differing reports of the extent of the toxic nature of these particles owing to their different modifications, surface functional attachments, shape and size [10,11]. Moreover, the metallic nature of the metal derived NPs and the presence of transition metals encourages the production of reactive oxygen species (ROS) leading to oxidative stress [12,13].

Although some scientists consider NPs as nontoxic, there are other studies reporting the toxic effects of NPs [14-16]. While some NPs may appear to be nontoxic, other cellular mechanisms such as cell signaling and other normal cellular functions may be disrupted and are currently undergoing further investigation [17,18]. The toxicity of NPs is being addressed by a number of standardized approaches with in vitro, in vivo as well as detailed genomic or biodistribution studies [18]. In addition, it has been shown that NPs may produce in vitro toxicity in some cell-based assays, but not in others. This may be a result of interference with the chemical probes, differences in the innate response of particular cell types, or other factors, a point to be considered when GNPs are used as carriers for the delivery of drugs and in gene therapy [19,20].

While nanotoxicity research is now gaining attention, little is paid to the effect of size and period of NPs mainly to their distribution and alterations in the tissue [21,22]. In the present study, an attempt has been made to address the possible histological alterations in the renal tissues following exposure to GNPs and, if so, whether the potential nanotoxicity is related to the size of these particles and the time of exposure.

## Materials and methods

A total of 70 healthy male Wistar-Kyoto rats obtained from the Laboratory Animal Center (College of Pharmacy, King Saud University, Saudi Arabia). The rats nearly of the same age (12 weeks old) and weighing 220-240 gm of King Saud University colony were used. Animals were randomly divided into groups, 6 GNPs-treated rats groups and one control group. Following a period of stabilization (7 days), 10, 20 and 50 nm GNPs were administered intraperitonealy at the rate for 3 or 7 days as follows: Group 1: received infusion of 50 μl GNPs of size 10 nm for 3 or 7 days (n = 10); Group 2: received infusion of 50 μl GNPs of size 20 nm for 3 or 7 days (n = 10); Group 3: received infusion of 50 μl GNPs of size 50 nm for 3 or 7 days (n = 10); Group 4: received infusion of 100 μl GNPs of size 10 nm for 3 or 7 days; (n = 10); Group 5: received infusion of 100 μl GNPs of size 20 nm for 3 or 7 days (n = 10); Group 6: received infusion of 100 μl GNPs of size 50 nm for 3 or 7 days; (n = 10); Control group: received no GNPs (n = 10).

The rats were maintained on standard laboratory rodent diet pellets and were housed in humidity and temperature-controlled ventilated cages on a 12 h day/night cycle. All experiments were conducted in accordance with the guidelines approved by King Saud University Local Animal Care and Use Committee.

Fresh portions of both kidneys from each rat were cut rapidly, fixed in neutral buffered formalin (10%), then dehydrated, with grades of ethanol (70, 80, 90, 95 and 100%). Dehydration was then followed by clearing the samples in 2 changes of xylene. Samples were then impregnated with 2 changes of molten paraffin wax, then embedded and blocked out. Paraffin sections (4-5 um) were stained with hematoxylin and eosin (the conventional histological stain) according to Pearse [23]. Stained sections of control and treated rats were examined for alterations in renal tissue.

## Results and discussions

No mortality occurred in any of the experimental groups of the present investigation, and no alterations were observed in the appearance and behavior of GNPs treated rats in comparison with the control ones. In comparison with the control group, the following histological alterations were detected in the renal tissue of GNPs treated rats: The following tubular alterations due to GNPs intoxication appeared in the renal tissue of the treated rats.

**Figure 1:**
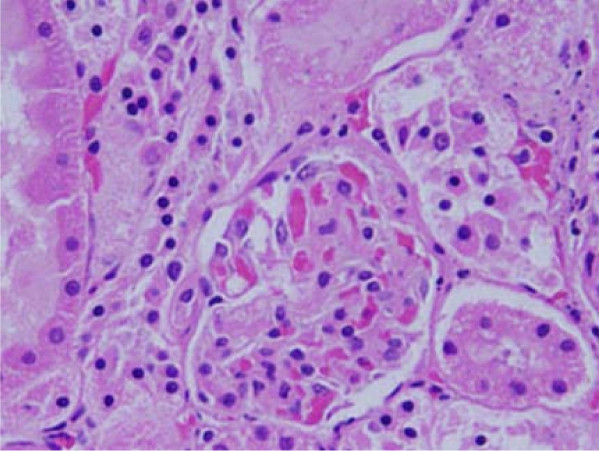
**Light micrographs of sections in the kidney of**: GNPs-treated rat received 100 μl of 10 nm particles for 3 days demonstrating dissociation of renal cells next to glomerular congested glomerulus.

GNPs produced occasional glomerular congestion in the rats exposed to 10 nm or 20 nm particles for 7 days but not in the glomeruli of the rats exposed to 50 nm (Figure 1). Hypercellularity and mesangial proliferation or glomerular basement membrane thickening was not detected in the glomeruli of all GNPs treated rats. Occasional dilatation of glomerular tuft blood capillaries was observed. These little alteration showed by the glomeruli might be due to the glomerular basement membrane which forms a barrier that prevents nanoparticles accumulation. Terentyuk et al., 2009 [24] have reported proliferation of epithelial cells of Bowman's capsule by GNPs where 15 nm particles showed more effect than larger ones.

### Dissociation of renal cells

dissociation of renal cells mainly next to glomerular congested glomeruli was seen (Figure 1). This alteration might indicate that GNPs affect renal cells adhesion and induces cell-cell junction disruption. According to Inumaru et al., 2009 [25] oxidative stress is a crucial factor to induce cell-cell dissociation.

### Renal tubules necrosis

**Figure 2:**
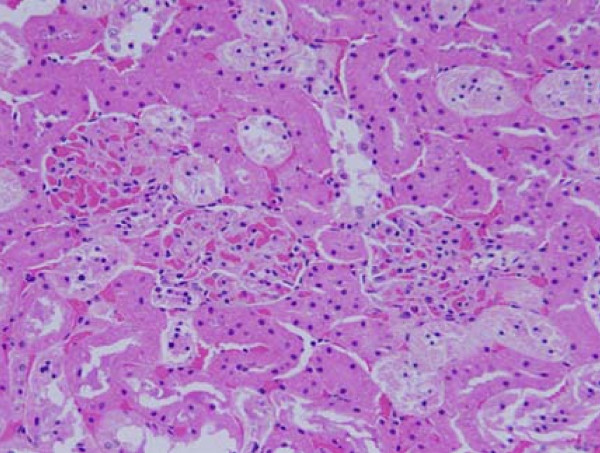
**Light micrographs of sections in the kidney of**: GNPs-treated rat received 50 μl of 20 nm particles for 3 days demonstrating renal tubules necrosis.

**Figure 3:**
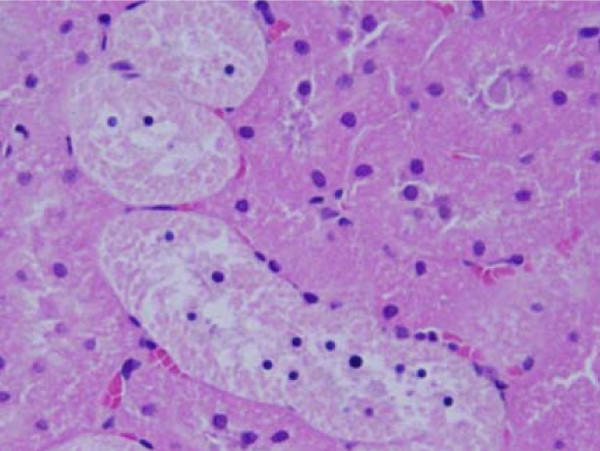
**Light micrographs of sections in the kidney of**: GNPs-treated rat received 100 μl of 10 nm particles for 7 days demonstrating renal tubules necrosis.

cellular necrosis was observed in the renal proximal tubules of GNPs-treated rats. The degenerative tubules showed swelling cytolysis and thinning or absence of the proximal tubular brush border and tubular irregularity (Figures 2 and 3). This alteration was more prominent in the cortex than the medulla and was accompanied with inter-tubular blood capillaries congestion and accompanied cytoplasmic eosinophilia in some of the necrotic proximal convoluted tubules. Tubular necrosis was detected in the renal tubules of rats exposed to 20 nm size particles and to lesser extent with 10 nm ones but was not seen with those exposed to 50 nm size particles. Necrosis was more prominent in the kidneys of the rats received 100 μl than 50 μl and less in 3 days than ones exposed to 7 days. Necrosis might be followed by organelles swelling specially mitochondria, endoplasmic reticulum and rupture of lysosomes before shrinking and dissolution of renal cells nuclei [26].

### Inflammatory cells infiltration

**Figure 4:**
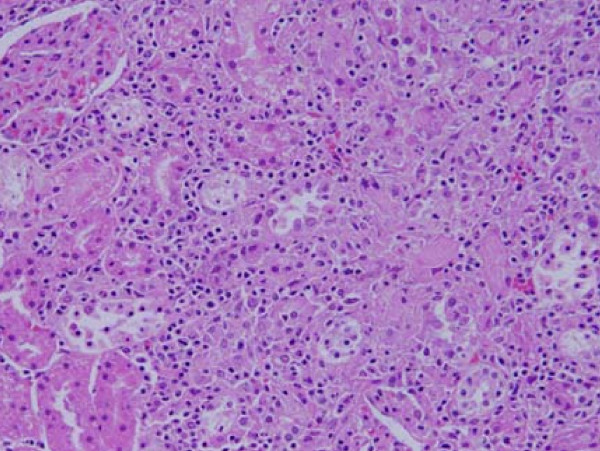
**Light micrographs of sections in the kidney of**: GNPs-treated rat received 100 μl of 20 nm particles for 7 days demonstrating inflammatory cell infiltration.

Inflammatory cells infiltration was seen in renal tissue of some GNPs treated rats. The infiltrate cells were mainly neutrophils and mononuclear cells (Figure 4). This infiltration was more after 7 days of administration and in rats received 100 μl than those received 50 μl but less prominent in the kidneys of rats exposed to 50 nm particles. The appearance of inflammatory cells in the renal tissue may suggest that GNPs could interact with proteins and enzymes of the renal interstitial tissue interfering with the antioxidant defense mechanism and leading to reactive oxygen species (ROS) generation which in turn may imitate an inflammatory response [27]. Yen et al., 2009 [28] have reported an induction of more immunological responses with smaller GNPs than the larger ones.

### Interstitial alterations

**Figure 5:**
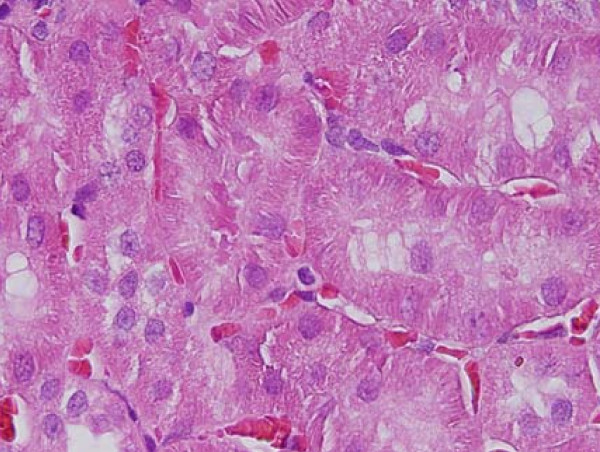
**Light micrographs of sections in the kidney of**: GNPs-treated rat received 100 μl of 10 nm particles for 7 days demonstrating intertubular blood capillaries dilatation.

**Figure 6:**
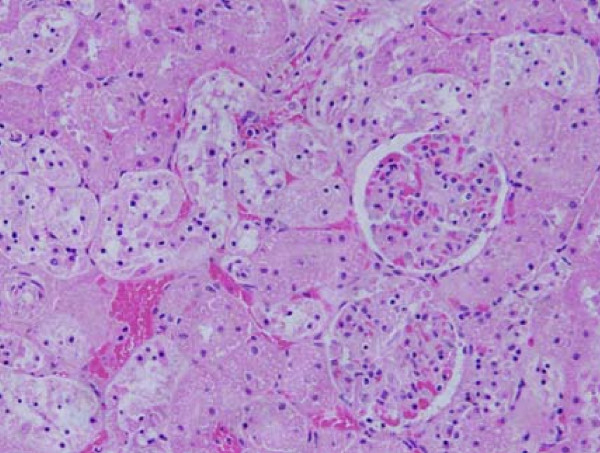
**Light micrographs of sections in the kidney of**: GNPs-treated rat received 100 μl of 20 nm particles for 7 days demonstrating intertubular hemorrahage.

The kidney of the GNPs treated rats showed the following interstitial alterations: 1) **Intertubular blood capillaries dilatation**: the kidneys of GNPs treated rats showed occasional inter-tubular blood capillaries dilation in rats received 100 μl of 10 or 20 nm particles for 7 days (Figure 5). This dilatation might be due to the decrease in the vascular resistance of the renal tissue induced by GNPs; 2) **Intertubular hemorrahage**: Focal intertubular hemorrhage was seen in the kidneys of rats exposed to 100 μl of 10 or 20 nm particles for 7 days (Figure 6).

**Figure 7:**
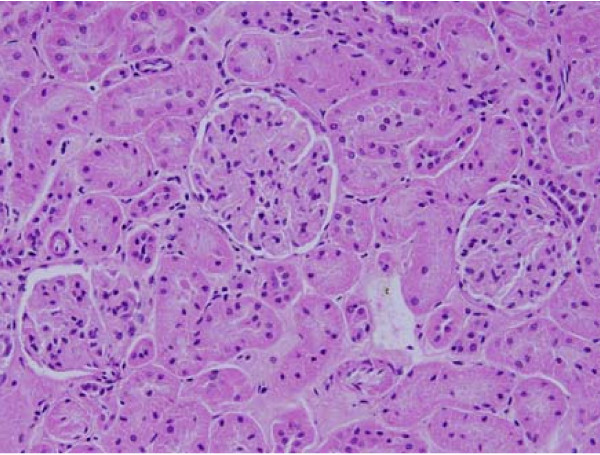
**Light micrographs of sections in the kidney of**: GNPs-treated rat received 50 μl of 50 nm particles for 3 days demonstrating normal glomerular structe.

**Figure 8:**
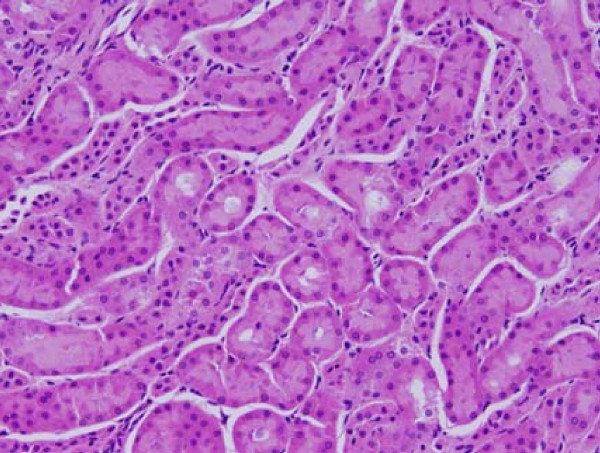
**Light micrographs of sections in the kidney of**: GNPs-treated rat received 100 μl of 50nm particles for 7 days demonstrating normal renal tubules.

The loop of Henle showed very little or no alterations due to GNPs intoxication while occasional cytomegaly and luminal hyaline casts were the only alterations observed in the collecting tubules. The renal tissue of the rats received 50 or 100 μl of 50 nm GNPs for 3 or 7 days showed little or no alterations while none of the above alterations were observed in the renal tissue of any member of the control group (Figures 7 and 8). Studies indicated that distribution in the inner organs is size dependent [24].

The results of the present work showed that the cortex is more affected than medulla due to treatment with GNPs. This could be partly due to uneven distribution of these particles in the tissue of the kidney where about 90% of the total renal blood flow enters the cortex via the bloodstream. Accordingly, a relative high concentration of particles might reach the cortex via the bloodstream than that would enter the medulla.

The findings of the present study indicate that the potential toxicity of GNPs is dependent upon the size of these particles. These findings are in line with the reports of Pan et al., 2007 [29] where cell lines where smallest GNPs demonstrated greatest toxicity compared to larger ones. Similar conclusion has been reported by Jong and Borm [30] when GNPs were injected intravenously into mice. This might be due to the fact that as size of a particle decreases, its surface area to volume ratio increases, resulting in increased surface reactivity with a tendency for toxicity even if the material is relatively inert in a bulk form [31-33]. Also, several studies indicate that the distribution of GNPs in the body tissue is size dependent [34,35].

## Conclusions

Histological alterations by GNPs exposure as shown in the results of the present work could be an indication of injured renal tissue due to GNPs toxicity that become unable to deal with the accumulated residues resulting from metabolic and structural disturbances caused by these particles. One might conclude that these alterations are size-dependent with smaller ones induced more damage to renal tissue with relation with the time exposure of GNPs. This might be due to earlier accumulation of the larger NPs in the tissue while the smaller ones stay much longer in the bloodstream due to recirculation.

The appearance of renal cells cytoplasmic degeneration and nuclear destruction may suggest that GNPs interact with proteins and enzymes of the renal tissue, interfering with the antioxidant defense mechanism and leading to generation and accumulation of the reactive oxygen species (ROS) which in turn may produce inflammatory response and mitochondrial destruction inducing stress in the renal cells to undergo atrophy, necrosis and programmed cell death.

More histomorphologcal, histochemical and ultrastrucural investigations are needed to correlate the biomedical application of GNPs with their therapeutic and diagnostic use in correlation with the size, chemical composition, surface charge, solubility and surface structure of these particles.

## Competing interests

The authors declare that they have no competing interests.

## Authors' contributions

MAKA and BMJ have analyzed data, interpreted and written the final draft of this manuscript. The animal model used in this study was obtained from the Laboratory Animal Center (College of Pharmacy, King Saud University, Saudi Arabia). Dr. MAKA has conceived the study and its design and obtained research grants for this study. Moreover, both authors have read and approved the final manuscript.
